# EARLY-LIFE EDUCATION CONTEXTS AND LATE-LIFE COGNITIVE STATUS: TOWARD IDENTIFYING MODIFIABLE RISKS

**DOI:** 10.1093/geroni/igae098.4169

**Published:** 2024-12-31

**Authors:** Kimson Johnson

**Affiliations:** University of Michigan, Ann Arbor, Michigan, United States

## Abstract

The complexity of K-12 school environments arises from their diverse student compositions, historical and geographical contexts, all of which may influence late-life cognition. Despite this, the long-term impacts of these factors on cognitive status in later life are not well understood. This study utilized data from the Health and Retirement Study (HRS) 2015-2017 Life History Mail Survey (N = 6,980; Mage = 72.5) to investigate how K-12 educational contexts—specifically school racial composition (White, Black, Hispanic, Other, and Mixed) and school setting (Public, Private, Mixed)—affect cognitive status in late life. Cognitive status was assessed in the 2020 HRS biennial wave using the Langa-Weir algorithm, classifying participants into Normal, Cognitive Impairment but No Dementia (CIND), and Dementia categories. After adjusting for sociodemographic and childhood factors, multinomial logistic regression analysis revealed that participants who attended predominantly Black K-12 schools had a higher risk of CIND and Dementia (RRR=2.28, 95% CI: 1.36, 3.80 and RRR=2.09, 95% CI: 1.08, 4.07, respectively). In contrast, those who attended private and mixed schools had a lower relative risk for CIND compared to public school attendees (RRR=0.43, 95% CI: 0.27, 0.68 and RRR=0.54, 95% CI: 0.37, 0.79). Additionally, participants from mixed school settings exhibited the lowest relative risk for dementia (RRR=0.51, 95% CI: 0.30, 0.88). Rural education was associated with a higher relative risk for CIND (RRR=1.26, 95% CI: 1.02, 1.58) compared to urban education. These findings highlight the need for targeted interventions and policies to create equitable educational environments that support cognitive health in later life.

